# Laparoscopy‐assisted vasovasostomy for post‐herniorrhaphy vas deferens obstruction

**DOI:** 10.1002/iju5.12150

**Published:** 2020-02-24

**Authors:** Masahiro Uchida, Shuichi Iida, Kazuhiko Hoshi, Kosuke Kojo, Haruki Tsuchiya, Kazumitsu Yamasaki, Jun Miyazaki, Teruaki Iwamoto

**Affiliations:** ^1^ Reproduction Center International University of Health and Welfare Hospital Nasushiobara Japan; ^2^ Department of Urology Tsukuba Gakuen Hospital Tsukuba Japan; ^3^ Suzuki Memorial Hospital Iwanuma Japan; ^4^ Prefectural Art Museum Mae Ladies Mental Clinic Shizuoka Japan; ^5^ Department of Urology University of Tsukuba Tsukuba Japan; ^6^ Department of Urology School of Medicine International University of Health and Welfare Narita Japan; ^7^ Division of Male Infertility Sanno Hospital Center for Human Reproduction International University of Health and Welfare Tokyo Japan

**Keywords:** herniorrhaphy, laparoscopy, vas deferens, vasovasostomy

## Abstract

**Introduction:**

Repair of obstructive azoospermia caused by childhood herniorrhaphy may be difficult. Therefore, intracytoplasmic sperm injection using testicular sperm is performed. However, vasovasostomy combined with laparoscopic surgery is challenging.

**Case presentation:**

A 42‐year‐old man underwent inguinal hernia repair at age 3. He had normal testicular size, azoospermia, normal hormone levels (follicle‐stimulating hormone, luteinizing hormone, and testosterone), absence of Y chromosome micro deletion, and karyotype:46XY, t(1:21)(p34.1:q22.3). He was diagnosed with obstructive azoospermia. Repeated intracytoplasmic sperm injections using testicular sperm resulted in miscarriages. Vasovasostomy combined with laparoscopic surgery was subsequently performed. Postoperative semen analysis result was almost normal. After intracytoplasmic sperm injection of ejaculated sperm, his wife got pregnant.

**Conclusion:**

Even if patients have chromosomal abnormalities, performing microsurgical re‐anastomosis first is recommended. To our knowledge, this is the first case of a laparoscopy‐assisted vasovasostomy for post‐herniorrhaphy vas deferens obstruction in Japan.

**Abbreviations & Acronyms**
ICSI: intracytoplasmic sperm injection
IH: inguinal herniorrhaphy
MR: microsurgical reconstructive procedures
OA: obstructive azoospermia
TESA: testicular sperm aspiration
Vas: vas deferens
V‐V: vasovasostomy

**Keynote message**

We report a case of laparoscopy‐assisted V‐V for OA after IH repair. Despite repeated attempts of ICSIs using testicular sperm, pregnancy did not occur. Laparoscopy‐assisted V‐V was subsequently performed. After ICSI of ejaculated sperm, the wife got pregnant and delivered a healthy boy.

## Introduction

Vas obstruction caused by IH repair is a common cause of seminal tract obstruction.^1^ The incidence of injury is 0.3–7.2% in adult IH repair, but reaches 27% in patients with a history of IH repair.^2^, ^3^ In Japan, its incidence rate in 2000 was 27%, which decreased to 9% in 2013,^2^, ^4^, ^5^ and the cause of iatrogenicity has decreased.

For vas obstruction treatment, Sheynkin *et al*. reported a total patency rate of 65% and a pregnancy rate of 39% after MR.^6^ However, in 56.7% of OA cases, the distal end of the vas was found in the pelvic cavity.^2^ This is believed to be due to the technical difficulties of MR. Although various surgical approaches have been suggested to bridge large vas defects,^2^ the length of vas defects rendered direct V‐V either impossible or dangerous because of tension.

Moreover, repair of OA caused by childhood herniorrhaphy may be difficult. Therefore, ICSI using testicular sperm is performed. However, V‐V combined with laparoscopic surgery is challenging, and no case has been reported in Japan. In our experience, inguinal vas obstruction was corrected by laparoscopic harvesting of the pelvic vas to be anastomosed microsurgically through the internal inguinal ring, thus bypassing the obstructed inguinal vas. This technique should provide enough length of the vas for a tension‐free anastomosis, and laparoscopy provides easier access without difficult dissection through the site of the previous hernia repair. Herein, we present our experience of laparoscopy‐assisted V‐V for post‐herniorrhaphy vas obstruction.

## Case presentation

A 42‐year‐old man underwent IH repair at age 3. His wife was 32 years old. He was diagnosed with OA. ICSI was performed repeatedly using TESA for infertility treatment at a hospital, but the wife did not become pregnant. Later, he decided to have V‐V. Testicular size was normal (right 20 mL, left 24 mL). The epididymis swelled slightly, and bilateral vas deferentia were dilated. Serum follicle‐stimulating hormone, luteinizing hormone, estrogen, and testosterone levels were 2.67 IU/L, 2.03 IU/L, 21 pg/mL, and 4.28 ng/mL respectively. Semen analysis revealed azoospermia. Chromosomal examination revealed 46XY, t(1:21)(p34.1:q22.3).

**Figure 1:**
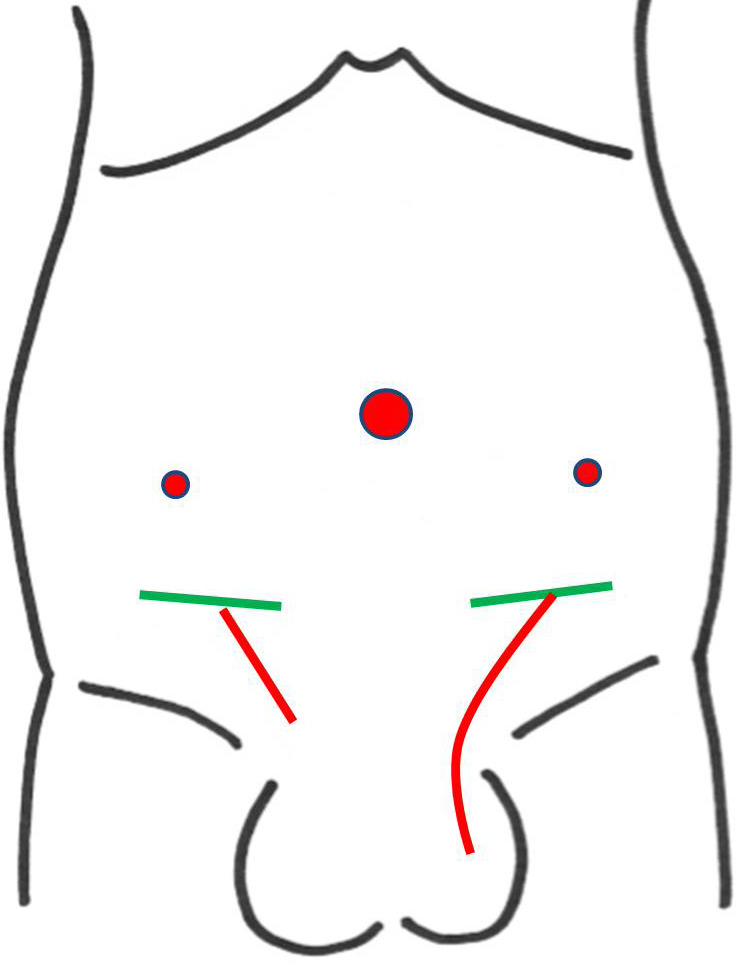
Schema of wound (red circles, laparoscopic port; red lines, wound of this operation; green lines, scar of IH repair).

**Figure 2:**
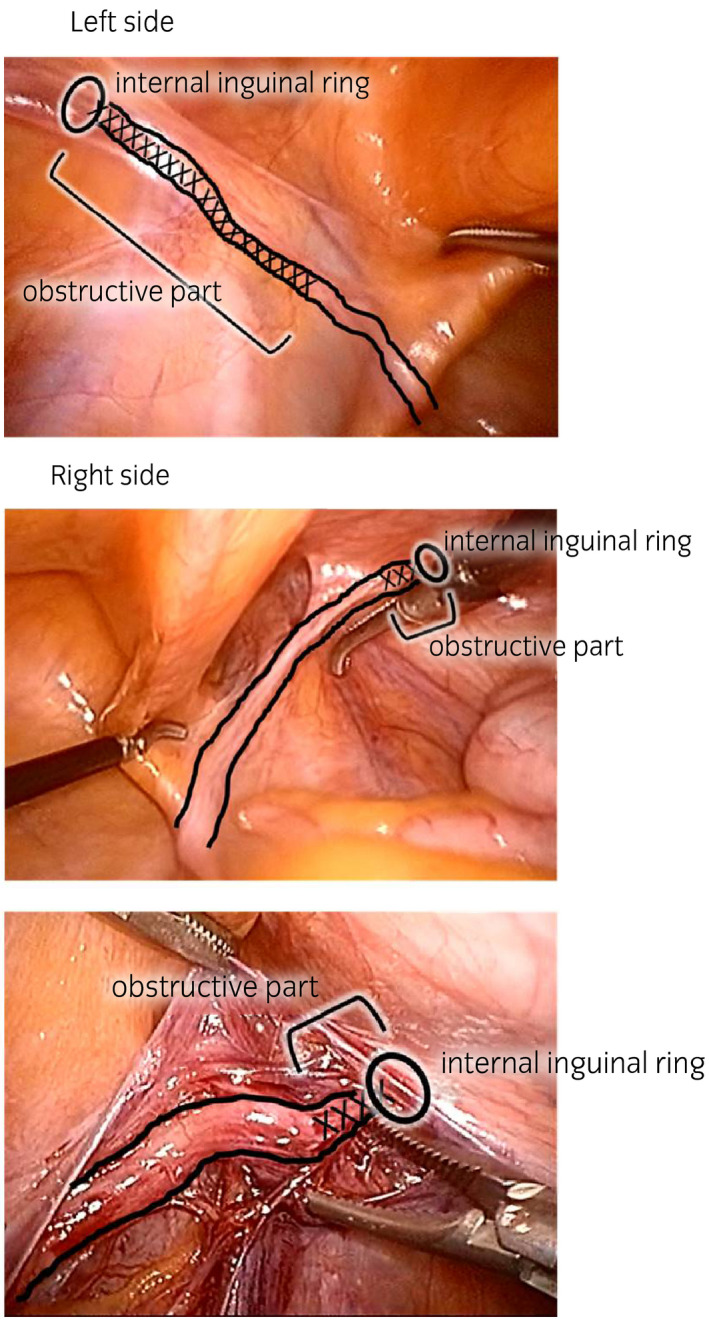
Obstruction of vas under laparoscopy.

**Figure 3:**
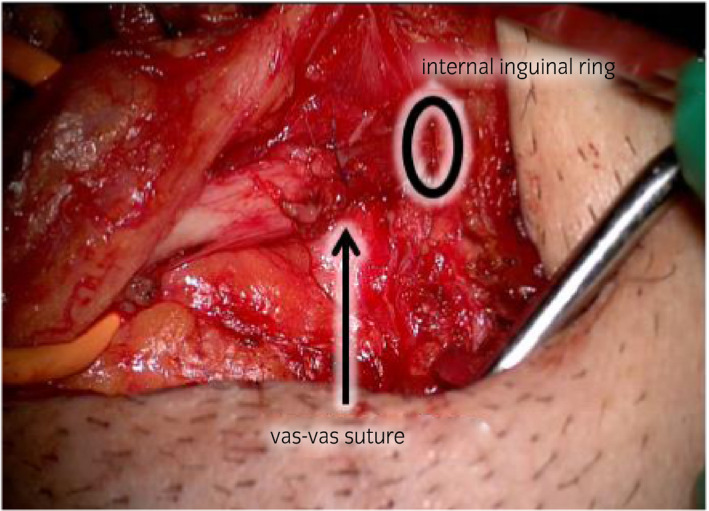
After V‐V under microscopy.

**Figure 4:**
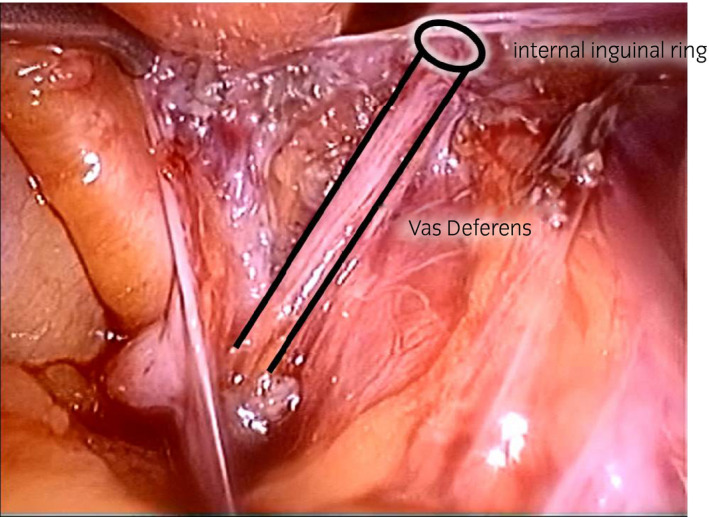
After V‐V, the vas observed under laparoscopy.

First, testicular sperm extraction was performed for the left testis for sperm cryopreservation. Then, we exfoliated the spermatic duct toward the cranial side. We opened the left inguinal canal but could not identify the obstructed vas, so we performed laparoscopy with head‐down position. A 10‐mm port was placed at the inferior umbilical crease, and the laparoscope was inserted. The abdominal pressure was 10 mmHg. Two 5‐mm ports were placed between the anterior superior iliac spine and umbilicus (Fig. 1). The peritoneum overlying the vas was incised on the left internal ring. With the laparoscope, the obstruction and vas defect were easily observed. Consequently, we judged that the left vas defect was too long to be repaired with V‐V. Furthermore, the right inguinal canal was opened; since the laparoscope was already inserted, the obstruction distance was 1 cm (Fig. 2). A 5 cm length of the distal vas could be freed from the surrounding structures under laparoscopy. The vas was then drawn out from the internal ring, enabling V‐V under microscopy. V‐V was performed with mucosal and muscle layers, which were sutured by 10‐0 and 9‐0 nylon sutures respectively (Fig. 3). Finally, we checked the anastomosed part of the vas for tension using laparoscopy (Fig. 4). The peritoneum was not closed. The left and right wound lengths were 10 and 5 cm respectively (Fig. 1). Testicular histology was “only a spermatozoa was present, and the Johnsen score was 8.” Two months later, sperms were observed in the ejaculated semen. Semen analysis at 6 months post‐operation was 28.1 × 10^6^/mL, with progressive motility rate of 38.0%. However, natural pregnancy was not achieved at 12‐month post‐operation, so they chose ICSI with ejaculated sperm.

## Discussion

Herniorrhaphy is one of the most common causes of iatrogenic vas obstruction.^1^ Vas injury following IH repair can be caused by cutting, crushing, or overstretching of the vas.^3^ Treatment of iatrogenic injuries is usually a challenging problem. Compared with vasectomy reversal, procedures performed after IH repair are more difficult, with lower success rate.^7^, ^8^, ^9^ Obstruction occurs commonly in inguinal lesion or in the retroperitoneal cavity. In the latter, microsurgical anastomosis might be possible if the obstruction site was directly observable by a cranial extension of the incision line. However, this method may result in a larger incision with the destruction of the upper wall of the inguinal canal, which might be a risk factor for hernia recurrence. Laparoscopic observation and releasing of the pelvic vas are reported to be safe and effective methods with minimum incision, after which tension‐free microsurgical anastomosis can be performed within the inguinal canal.^10^ Recently, Kuang *et al*.^11^ reported that robot‐assisted V‐V has been easily performed, but in Japan, it is not performed because it is not covered by the Japanese public health insurance.

There is controversy of whether we should proceed with MR or ICSI using TESA^12^ or percutaneous epididymis sperm aspiration^13^ to achieve pregnancy after surgery for OA. Many patients are currently undergoing ICSI without trying MR. This may be due to several disadvantages of MR after IH, such as the operative technical difficulty, and sperm appearance and birth rate after MR are lower than those of MR for OA.^2^, ^14^ Shiraishi *et al*. emphasized that the benefits of surgery, natural pregnancy, and cost‐effectiveness in the MR group were higher than those in the ICSI group. Compared with ICSI, recent data suggest that MR done by a skillful microsurgeon is cost‐effective.^4^, ^15^, ^16^, ^17^

In our case, the physician first performed ICSI because the husband’s chromosomal analysis revealed autosomal translocations. However, the wife had frequent miscarriages. Despite surgical success and normalized semen findings, natural pregnancy was not achieved for 1 year. ICSI with ejaculated sperm was selected; fortunately, the wife became pregnant and gave birth. The successful birth after MR cannot be clearly defined because chromosome examination of fertilized eggs was not performed, but we would like to interpret that the fertilized eggs had normal chromosomes or balanced reciprocal translocations, and the success of birth could be attributed to the benefits of the maturation process during the passage of the testicular sperm through the epididymis.^18^

Even if OA patients have chromosomal abnormalities, it may be acceptable to perform MR prior to ICSI.

## Consent

Consent was obtained from the patient.

## Conflict of interest

The authors declare no conflict of interest.
